# Use of Artificial Intelligence-Based Strategies for Assessing Suicidal Behavior and Mental Illness: A Literature Review

**DOI:** 10.7759/cureus.27225

**Published:** 2022-07-25

**Authors:** Nighat Z Khan, Muhammad Ali Javed

**Affiliations:** 1 Transitional Year Medicine, Mercy Hospital St. Louis, St. Louis, USA; 2 Critical Care Medicine, Mercy Hospital St. Louis, St. Louis, USA

**Keywords:** mental disorder, mental health, suicidal thoughts, psychiatry, machine learning, natural language processing, behavior, suicide, artificial intelligence

## Abstract

Mental illness leading to suicide attempts is prevalent in a large portion of the population especially in low and middle-income nations. There remains a significant social stigma associated with mental illness that can lead to stigmatization of patients. Hence, patients are reluctant to communicate their problems to health care providers. Physicians have difficulty in timely identification of patients at risk for suicide. Novel and rigorously designed strategies are needed to determine the population at risk for suicide. This would be the first step in overcoming the multitude of barriers in the management of mental illness. Clinical tools and the use of electronic medical records (EMR) are time intensive. Recently, several artificial intelligence (AI)-based predictive technologies have gained momentum. The aim of this review is to summarize the recent advances in this landscape.

## Introduction and background

At any point in time, a large proportion of the population is affected with mental illness. As a result, prevention and timely management of mental illness has recently become a global health priority [1]. Mental illnesses and suicide attempts are becoming a huge health burden worldwide [1]. According to the World Health Organization (WHO), in 2016 the suicide rate was estimated to be 10.6 per 100,000 population, and the majority (80% of these suicides) occurred in low- and middle-income nations [2]. Recently, a United States (US) report mentioned suicide as the tenth most common cause of mortality in adults [3]. In 2019 in the US alone, approximately 1.38 million suicide attempts, and 47,511 deaths were reported [3].

Mental illness is caused by an interplay of various risk factors. The combination of the absence of appropriate medical treatments and the lack of adequate family support, frequently aggravates mental illness into suicidal behavior [4]. Individuals at risk of suicide frequently do not communicate their problems or challenges to their physicians nor their communities because of generalized societal disapproval and stigma, and a history of forced medical treatments [4]. In addition, individuals with a psychiatric illness (including a large proportion of individuals who ultimately commit suicide) have poor insight into their mental state, and thus do not self-identify as being in any kind of danger [4]. Both these problems (lack of reporting and the poor insight) are further exacerbated by the difficulty that physicians and other providers have with the timely identification of people at risk for suicidality [4]. Therefore, it is critical that physicians are able to conduct suicide risk assessments on high-risk patients. These assessments themselves are challenging because they depend on a patient’s location, the availability and access to health care, and the patient's overall suicide plan (and its vocalization) [5]. The recent coronavirus disease 2019 (COVID-19) pandemic has only exacerbated this suicide risk, especially in the elderly [6]. Based on recent literature, two major factors for this increased suicidality in the elderly are, the self-isolation required with COVID-19 and the loss of connections to family members and the outside world in general [6].

Existing clinical tools used for suicide risk assessment tend to be time-intensive, cost-prohibitive, and usually need practitioner guidance for administration [7]. Novel and rigorously designed strategies are required to determine the risk of suicide, and to successfully overcome economic, clinical, and infrastructure barriers. These are starting to include artificial intelligence (AI)-based technologies [8]. Responding to these requirements for more practical mental status evaluations, various healthcare technologies and digital applications have recently started gaining popularity [9]. Previous studies had largely focused on the adoption of electronic medical records (EMR) to diagnose the mental well-being of an individual. A major limitation with this method is that it is less accurate and has decreased efficiency, compared to novel digital diagnostic tools. Thus, a computerized algorithm (if available) would be ideal to assess mental health status based on the clinical history and electronic health records (EHRs) of patients. This algorithm would ideally be able to classify a patient's risk simply based on their symptom severity [9]. It is hoped that with the employment of AI and machine learning (ML), new prospects will be available to guide early suicide risk prediction, classify mental health status, and improve suicide preventive interventions [10].

Several AI-based prediction technologies are already gaining popularity in medicine. These include the detection of medical errors, enhanced patient safety parameters, and the assessment of chronic conditions [8]. We believe that the use of AI in mental health will be welcomed by practitioners and patients alike.

The main aim of this systematic review is to characterize and then summarize the recent advances in AI [both with machine learning (ML) and natural language processing (NLP)], specifically for suicidal behavior evaluation and mental disorders diagnosis. The techniques and tools that are currently being used in mental health practices will be defined in methodological and technical terms. These include techniques used for diagnosis and prognosis, treatment adherence, adverse events, risk factors, and finally, the impact of psychotherapy. We will elaborate the AI techniques used for the assessment of suicidal behavior and mental health. We also intend to summarize the successful use of AI tools in mental health settings such as diagnosis, suicide prediction, and identification of suicide risk factors.

## Review

Methods

Search Strategy

A computerized literature search was performed in MEDLINE (PubMed), the Cochrane Library, and Google Scholar databases from 2010 till Dec 2021. The literature search included the following keywords: Artificial intelligence, suicide, behavior, natural language processing, machine learning, psychiatry, suicidal thoughts, mental health, and mental disorder.

Inclusion Criteria

Based on the availability of the full-text articles, the relevant studies were reviewed to ensure that they met the inclusion criteria as follows:

1) Studies published in the English language.

2) Original journal research articles limited to randomized controlled trials (RCTs) and reviews.

3) Studies including AI-driven models with ML and NLP methodologies.

4) Studies including patients with risk of suicidal behavior, suicide attempts, suicide ideation, and suicide-related death.

5) Studies with AI-based technology for evaluation of mental health status.

Exclusion Criteria

1) Meta-analyses, editorials, letters, abstracts, comments, and book chapters were excluded.

2) Studies with improper randomization protocols (improper allocation to study groups).

3) Studies published in languages other than English (n=4).

4) Studies where the mental health status was not clearly demarcated or those that did not have essential data (n=30).

Study Selection

**Figure 1:**
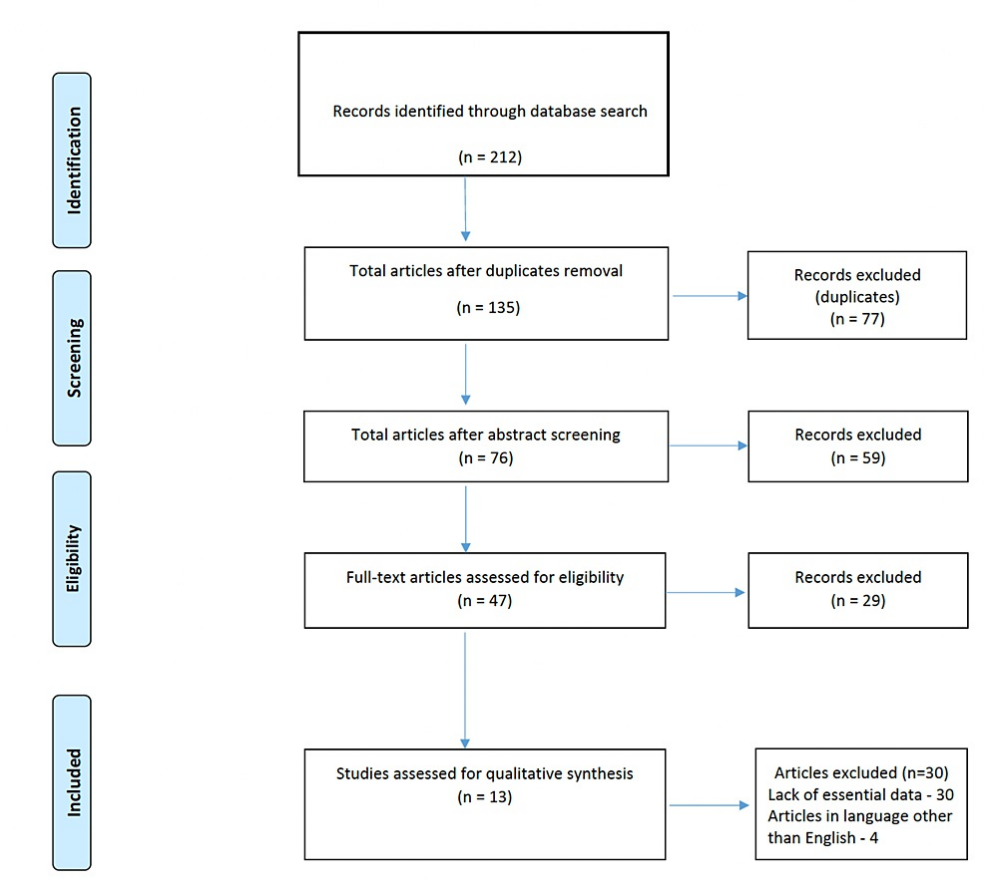
Flow diagram illustrating the literature search and selection criteria (according to PRISMA, Preferred Reporting Items for Systematic Reviews and Meta-Analysis)

The relevant studies were selected in two stages after the search strategy was initiated. First, the authors extracted the required data based on the eligibility criteria (as described above). All available titles and abstracts were identified and read to ensure that the included studies met the inclusion criteria. The full-text articles were then obtained and read in the second stage. Science Citation Index (http://www.isinet.com) was also used to find papers that had cited these articles, as well as to find potentially relevant articles for a secondary review. Finally, based on the inclusion criteria, studies were included in this review, as detailed in Figure 1.

Data Extraction and Quality Assessment

Studies that fulfilled the inclusion criteria were processed for study details and outcome data. The primary focus of the review was the assessment of suicide, and the role of AI tools in assessing suicidal behavior and mental status.

Each study was analyzed, and the information was extracted as highlighted in Table 1. The process of including studies was characterized into different categories such as name of author, year, study design, AI tools, location of study, and study findings.

**Table 1:** Demonstrates the key characteristics of the included studies: AI tools, study design, location, findings, and clinical significance. SI – suicidal ideation; NLP – natural language processing; EMR – electronic medical records; EHR – electronic health record; AI – artificial intelligence; ML – machine learning; SAIPH – Suicide Artificial Intelligence Prediction Heuristic; DSM – Diagnostic and Statistical Manual of Mental Disorders; ANN – artificial neural network; MMI – major mental illness; PPV – positive predictive value; LR – logistic regression.

S. No	Author	Subjects	AI Tools	Study design	Location	Findings	Clinical significance	References
1.	Carson et al. (2019) [9]	Identification of suicidal behavior among hospitalized adolescents admitted in the psychiatric department.	NLP of EHR	Survey	USA	NLP model showed specificity, sensitivity, accuracy, and PPV of 0.22, 0.83, 0.47, and 0.42, respectively. The model also identified key terms related to suicidal attempts that were closely associated to family members, suicidal thoughts, psychotropic medications, and psychiatric disorders.	Use of NLP and ML showed modest success in determining suicide attempts in the psychiatric department.	8
2.	Cook et al. (2016) [11]	Assessment of psychiatric symptoms and SI among adult psychiatric inpatients discharged from the hospital	NLP and ML	Qualitative study	Madrid	The NLP-based model demonstrated a better performance for assessing SI including specificity, PPV, and sensitivity with values of 0.57, 0.61, and 0.56, respectively, compared to the traditional models where the values were 0.62, 0.73, and 0.76, respectively. The outcome was overall positive for NLP-based models in assessing heightened psychiatric symptoms.	NLP-based models demonstrated better outcomes in assessing general mood. Have a higher predictive value in determining suicide risk and psychological distress simply based on general questions.	10
3.	Glaz et al. (2021) [12]	Understanding the significance of studies that used AI techniques for determining mental status.	NLP and ML models	Qualitative study	-	Assessed 327 articles. This review highlighted that language-specific features should be incorporated to enhance the usefulness and performance of different NLP methods.	ML and NLP proved a significant tool in determining the state of mental health. These tools should be used to gain perspective in clinical practice.	11
4.	Cohen et al. (2020) [13]	Use of AI to assess the risk of suicide by use of a mobile application by patients undergoing therapy sessions. Parameters used: Scores of suicidality and depression standardized scale, language samples, and impression of client’s mental status according to the therapist.	NLP and ML models	Qualitative study	USA	Interviews were collected and the therapist evaluated patients’ risk for self-harm or suicide. Findings demonstrate better outcome with logistic regression and vector machines with an accuracy of 0.76 and 0.75, respectively. These models have good discriminative ability allowing improved overall performance.	ML methods can help improve mental health outcomes during therapy sessions. They are effective in identifying mental health based on language samples and voice collections.	12
5.	Fernandes et al. (2018) [14]	Categorize the severity of suicide ideation and investigate the number of suicide attempts reported in a clinical psychiatric database.	NLP and hybrid ML	Scientific report	London	A hybrid model was successful in identifying the number of suicide attempts, with a precision of 82.8%, and a sensitivity of 98.2%. The precision for correct identification of suicidal ideation was 91.7%.	Algorithm based on a dual model of NLP and hybrid ML was able to successfully extract suicide behavior factors, and clinical data from a psychiatric database.	21
6.	Oh et al. (2020) [15]	Determine the different factors related to suicidal ideation and create AI models to predict risks.	ML	Cross sectional	Korea	ML models demonstrate better prediction performance than conventional LR model. AI models outperform traditional models.	ML approach had better performance in predicting suicidal ideation than other models.	20
7.	Graham et al. (2019) [16]	Categorizing mental illness based on: EHR, social media platforms, brain imaging data, novel monitoring systems (e.g., mobile apps), mood rating scales	ML algorithms	Narrative synthesis	USA	ML algorithms useful in classifying mental health disorders (schizophrenia, depression, and suicidal ideation and attempts). Potential to help mental health clinicians differentiate mental health disorders more objectively than relying on traditional DSM-5 scores.	AI techniques can determine mental health outcomes effectively, helping mental health practitioners better understand mental disorders.	14
8.	Corke et al. (2021) [17]	Relationship between number of suicide risk factors included in an algorithm and strengthening of predictions in suicide prediction models.	ML	Review article	England	Results found a higher odds ratio when numerous risk factors for suicide were included in the ML-based studies. (P = 0.02)	ML can enhance the performance of predicting the risk of suicide by increasing the number of suicide risk factors evaluated. Superiority over other methods is yet to be confirmed.	18
9.	Kumar et al. (2020) [18]	Use of AI for the identification of self-harm (uncoded) in severe mental disorders (major depression, schizophrenia, schizoaffective disorder, and bipolar disorder).	ML	Narrative synthesis	USA	AI learning models had an agreement of 83.5% with traditional models. Also found that patients with MMI had the highest overall incidence of encountering coded self-harm.	ML displayed significant success in identifying self-harm visits.	19
10.	Roy et al. (2020) [19]	Based on Twitter data (publicly available) predicting the risk of suicidal thoughts in the future	Algorithm SAIPH	Qualitative study	Canada	A neural network model assessed tweets based on SI cases. AI-based random forest model enabled the prediction of suicidal ideation status. The model demonstrated an accuracy of 0.88 in differentiating suicidal ideation from controls.	SAIPH, as an algorithm has the potential to predict future SI. Can enable clinicians to use this tool for suicide screening and risk assessment.	13
11.	Yang et al. (2021) [20]	Investigating the risk of suicide among users of the website “Zou Fan Tree Hole” and conducting the suicide crisis intervention with high suicide risk (level 6-10).	Tree Hole Intelligent Agent (Artificial Intelligence Program)	Qualitative study	China	The “Tree Hole Action” served as a significant online tool to prevent 3629 potential suicides.	Tree Hole Action showed improved outcomes in monitoring suicide risk and interventions for online users. Example of coordination of AI, social forces, and mental health services to provide the required support to individuals at high suicide risk.	17
12.	Gong et al. (2019) [21]	Use of AI models to determine patterns and progression of depression according to fitted depression trajectories.	ANN		Southern California, Colorado & Washington	ANN model had a strong tendency to demarcate depression severity.	AI models proved helpful in the detection of patterns of depression, its progression, and changes in symptoms over time. Will help design a trajectory-based method for depression patients.	16
13.	Zhong et al. (2019) [22]	Classifying AI algorithm that helps predict the risk of suicidal behavior in pregnant women.	NLP in EHR	Review	Boston	NLP models were successful in improving the identification of suicidal ideation and suicidal attempts. The model was able to correctly predict suicide cases that increased from 125 to 1423, even though the PPV was similar to diagnostic codes of suicidal ideation.	NLP helped predict suicidal behavior by mining unorganized clinical notes. NLP was effective in predicting suicidal behavior in pregnant women by 11-fold.	15

Results

Selection of Studies

The process of retrieving and screening the studies which were included in this systematic review is shown in Figure 1. After an initial search, a total of 212 articles were identified. After removal of duplicates (77) there were 135 studies of interest remaining. These were screened by study titles and abstracts and only 76 studies were found to be randomized controlled trials (RCTs), reviews, and scientific reviews. Others (59) were meta-analyses, editorials, letters, abstracts, or comments. These 76 studies were critically evaluated and some of them had improper randomization protocols (improper allocation to study groups) whereby another 29 were excluded. This left 47 full-text articles that were reviewed in entirety for eligibility per inclusion criteria. Studies published in languages other than English (n=4) and studies where the mental health status was not clearly demarcated or those that did not have essential data (n=30) were excluded. Thus 13 studies which met all the inclusion criteria, were included in this review.

In these included studies, the different categories of participants included were:

1) Patients whose electronic health record data was available on authentic databases with their overall medical records.

2) Self-reported data obtained from social media platforms such as Twitter and Facebook.

3) Patients who were admitted to psychiatry departments.

Population

The collected data represents populations from various locations including the USA, Madrid, Southern California, Colorado, Washington, Canada, Boston, China, England, Korea, and London. The common features mentioned in majority of the studies were suicides, depression, psychiatric disorders, mood disorders, schizophrenia, schizoaffective disorder, and bipolar disorder.

AI Tools

A wide spectrum of AI tools was used in these 13 studies. A combination of NLP and ML was used in four studies [11-14]. ML as the exclusive AI technique was used in four studies [15-18], artificial neural network (ANN) was used in three studies [19-21], and NLP in two studies [9,22].

Clinical Outcomes

The effectiveness of AI techniques was initially recognized by Cohen et al. who reported that ML methods can improve mental health outcomes during therapy sessions [13]. As mentioned by Cook et al. [11], NLP and ML helped decipher the overall mood and mental status of patients. An algorithm based on dual model of NLP and hybrid ML resulted in excellent outcomes to extract suicidal behavior factors, and clinical datasets from a psychiatric database [14]. These combinations of AI techniques were successful in determining the suicide risk and the psychological distress seen among adult psychiatric inpatients when they are discharged from the hospital. Similar results were shown in three of the studies included [9,12,15]. Most studies included in this review demonstrate improved performance with an AI-based approach compared to previously used traditional models.

A study by Corke et al. (2021) mentioned that by increasing the number of suicide risk factors, ML can improve the performance of suicide risk prediction. However, its superiority over other methods has yet to be proven [17]. AI learning models had an overall agreement of 83.5% with the traditional models with ML displaying significantly better results in identifying self-harm visits [18].

When discussing recognition of risk of suicides based on information available on social media platforms, two studies mention the potential use of AI in correctly identifying this suicide risk. First, the Suicide Artificial Intelligence Prediction Heuristic (SAIPH) algorithm was able to accurately predict future suicidal ideation [19]. In the second study, similar results were demonstrated after adopting “The tree hole action” algorithm [20], which eventually played a significant role in preventing suicides. Graham et al. [16] report that ML and NLP prove to be useful in classification of various mental health disorders such as schizophrenia, depression, and suicidal ideation. Similarly, Gong et al. also observed better performance with an AI-based approach in assessing the patterns of depression compared to usual care [21]. Lastly, one study found that NLP was more effective in predicting suicidal behavior in pregnant women specifically [22]. Overall, most studies demonstrated higher sensitivity, specificity, and positive predictive values (PPV) with AI-based techniques when compared to conventional tools.

Discussion

AI-Driven Prediction

Despite the promising results of this review, the utility of identification of suicidal attempts is limited because of only modest sensitivity and low PPV. This is because of the reduced incidence of actual suicide attempts compared to the prevalence of suicidal ideation [23]. However, despite the sensitivity issue AI-based techniques should still be helpful, as EMRs in their current form, will continue to remain minimally predictive.

Suicidal Ideation and Risk of Suicides

Numerous studies have identified the role and importance of AI in predicting the risk of suicide attempts among adolescents. In a study, Jung et al. applied ML-based approach to 60,000 Korean adolescents determining the suicide risk according to previous suicide attempts and suicidal ideation. They analyzed 26 factors predicting suicide risk, and five different models including support vector machine, artificial neural network (ANN), logistic regression (LR), extreme gradient boosting, and random forest, all demonstrating an accuracy between 77.5% and 79% [24].

A study by Corke et al. (2021) mentioned that by increasing the number of assessed risk factors for suicide, ML can improve the performance of suicide risk prediction models. Its superiority over other methods, however, has yet to be proven. The risk factors for determining suicidal ideation include parameters such as the history of continuous depressed mood, stress awareness, alcohol abuse, educational status, unmet medical needs, socio-economic background, and severity of depression. Data has demonstrated that ML models (such as LogitBoost, and ANN) were more effective than the traditional LR model (0.867) in assessing the risk of suicide [17].

Another study by Yang et al. (2021) investigated the risk of suicide among users of the website "Zou Fan Tree Hole" which was engaged in providing suicide crisis intervention to people at high risk for suicide (level 6-10). The Tree Hole Intelligent Agent is an Artificial Intelligence (AI) Program that eliminates a large portion of superfluous information and allows easy interpretation of data contained on the website, Tree-Hole. "Tree Hole Action" project yielded better results in terms of suicide risk monitoring and intervention for online users of this website. This is an example of AI, social forces, and mental health services working in unison to provide the required support to people at the highest risk of suicide [20].

AI technology is currently showing immense utility when it comes to its implementation for predicting the risk of suicidal behavior in pregnant women. Recently the American College of Obstetrics and Gynecologists (ACOG) advised that physicians should screen patients for depressive symptoms during their perinatal period [25]. Zhong et al. (2019) reported that by mining the disorganized clinical notes, NLP was able to predict suicidal behavior successfully. NLP was found to be 11-times more effective in predicting suicide risk in pregnant women [22]. Hence, the implementation of an AI algorithm in EMR systems can help improve the ability of correctly identifying (and hopefully decreasing) suicidal behavior [25].

Prior studies have demonstrated that conventional methods of predicting suicides based on written questionnaires, surveys, or scales are insufficiently accurate, time-sensitive, and require active participation of the respondent [26]. AI technology bypasses these limitations and so is more likely to help people receive prompt support and earlier treatment.

Mental Illness: Use of AI Prediction in Mood Disturbances and Depressive Symptoms

Current evidence suggests that more than 80% of individuals who die from suicide suffer from some form of mental illness [23]. A study by Cook et al. (2016) demonstrated that NLP-based models perform better at assessing general mood. These methods also have better predictive value when predicting suicide risk and psychological distress. ML proved effective with a higher sensitivity and specificity in analyzing the mental state compared to when this is assessed by general questions or open-ended texts obtained directly from the patients. A study by Cohen et al. describes that during therapy sessions, ML methods can help improve mental health outcomes by analyzing language samples and voice recordings [13].

Although structured knowledge requires considerably longer time involvement from the respondent, when exploited by computational analytics like NLP, information obtained from free-text responses can serve as an effective tool in predicting suicidal behavior and suicidal risk factors. This technology might have a profound impact to highlight red flags in patients with high suicidal behavior. Ultimately, this will help physicians intervene early and provide the required support for an immediately suicidal patient [11].

Current AI models are being used to determine the patterns and progression of depression based on depression trajectories that have already been studied [21]. Gong et al. (2019) studied AI models that were useful in detecting depression patterns, progression of symptoms, and changes in symptoms over time. This type of data can aid in the development of a depression-specific trajectory-based methodology. Gunn et al. [27] studied 789 patients from 30 randomly selected family medicine practices, with a prominent impact on their PHQ-9 scores over a 40-week period. Depressive symptoms were measured every three months for a year. By using trajectory modeling, this study was able to suggest future directions for prognostication.

Mental Illness: Use of AI in Medication Adherence

A novel use of AI could be with medication adherence in patients. One such avenue is clinical trials where medication adherence is usually determined by pill counts, which can frequently be unreliable. This was evaluated in a study by Bain et al. (2017), where patients with schizophrenia who used an AI platform had a higher rate of medication adherence (90%) than those who used modified direct observed therapy (72%) [28]. Secondly, in the clinical setting, especially psychiatry wards and even outpatients, AI could be successfully employed for medication administration and compliance.

Predicting Mental Illness Based on Social Media Information

A systematic review by Pourmand et al. described youngsters disclosing suicide risk factors on social media including Facebook and Twitter rather than reaching out to a physician [29]. The authors suggest that these social media platforms could serve as a clinical aid in detection and decision-making [29]. Similarly, another cross-sectional study investigated suicidal ideation and behavior according to self-reported data, based on 1000 Twitter users tweeting about their suicidal thoughts [30]. These types of data suggest that social media sites such as Twitter and Facebook, if analyzed appropriately and systematically, could actually play a significant role in determining the outcome metrics of suicidal behavior. An ML classifier has been developed by Burnap et al. that is capable of identifying high-risk individuals on Twitter with an accuracy of 68-73% [31]. With the implementation of this tool, a subsequent 12-month follow-up study determined that the classification system was 85% accurate compared to trained human raters [30]. Individuals with schizophrenia are more likely to tweet about suicide [23]. These can easily be identified by using ML as it could be designed to target suicidal ideation and suicidal behavior phrases. Recently, Du et al. (2018) implemented such an ML-based approach and designed a convolutional neural network (CNN) with the potential to identify suicidal tweets [32].

Hopefully, these techniques will eventually pave the way for mental health experts to provide prompt treatment and intervention to those identified to be at a higher risk for suicide. Currently, the main challenge is the limited ability of AI algorithms to predict suicidal thoughts prior to their development as models only identify such tweets after someone expresses them. Thus, it is difficult to identify suicidal thoughts and the mental states of individuals who do not tweet about it.

Limitations

This study has several limitations. First, only 13 articles were included in this study which met the inclusion criteria. This limits the generalizability of this review. However, the intent of the review was to evaluate the currently available evidence for its quality, and to make potential recommendations for future research directions.

Secondly, posts containing depressive-sounding words could indicate a passing state of depression instead of a full-blown depressive episode. In online posts, social media users may sometimes over-express manifestations, or their remarks may sometimes be purely situational. As online posts frequently lack complete contextual information, this information can easily be misinterpreted. Thus, the true clinical utility of these data-rich platforms requires more careful understanding, and studies involving social media should follow quality-based methodological standards [14].

Third, the predictive ability of these studies is restricted to the features that were used as input for the ML models (e.g., clinical data, demographics, biomarkers, etc.). After adopting any AI models, performance metrics need a thorough understanding so that the practicality and relevance of the results can be ascertained.

Fourth, the majority of the included studies have been conducted in high-income nations. This demonstrates the readiness of adopting such advanced digital tools might be limited to certain regions and locations. The inclusion of low-income and middle-income nations is necessary to understand the global application of these methods and the role these new models can play on a global scale.

Lastly, although several of these studies identified and characterized the risk factors associated with mental illness, further research should also consider factors that improve understanding of the preventative aspects in mental health. This will enable us to gain novel insight into decreasing the incidence of mental disorders, suicidal thoughts, and suicidal attempts in general. Therefore, large datasets will be required to obtain high-quality information and to conduct mental health research for promoting mental hygiene and reducing the risk of suicide for a given population.

## Conclusions

Artificial intelligence is becoming a larger part of digital medicine, and it will certainly help with mental health research and clinical practice. To realize the full potential of AI, a diverse community of experts involved in mental health research and care, including data scientists, healthcare professionals, regulatory authorities, and patients, must all collaborate and communicate effectively. The best results for patients will be obtained from the collective efforts of these stakeholders, who will need to work as a team to develop robust algorithms.

Combining clinical and social media-driven suicide prediction tools, according to the findings of this review, could strengthen our ability to recognize those who are at high risk of committing suicide. Eventually, this will improve our potential to help save lives. The present review determines the current advances and the future potential of various AI-driven models in predicting mental illness and the risk of suicide. However, further studies are needed to determine the validity, applicability, and moral implications of using these tools in geographically and economically diverse populations.
